# How compensation breaks down in Parkinson's disease: Insights from modeling of denervated striatum

**DOI:** 10.1002/mds.26579

**Published:** 2016-02-18

**Authors:** Charlotte Amalie Navntoft, Jakob Kisbye Dreyer

**Affiliations:** ^1^ Department of Neuroscience and PharmacologyUniversity of CopenhagenCopenhagenDenmark

**Keywords:** D1‐receptor, D2‐receptor, postsynaptic compensation

## Abstract

The bradykinesia and other motor signs of Parkinson's disease (PD) are linked to progressive loss of substantia nigra dopamine (DA) neurons innervating the striatum. However, the emergence of idiopathic PD is likely preceded by a prolonged subclinical phase, which may be masked by a variety of pre‐ and postsynaptic compensatory mechanisms. It is often considered self‐evident that the signs of PD manifest only when nigrostriatal degeneration has proceeded to such an extent that putative compensatory mechanisms fail to accommodate the depletion of striatal DA levels. However, the precise nature of the compensatory mechanisms, and the reason for their ultimate failure, has been elusive. In a recent computational study we modeled the effects of progressive denervation, including changes in the dynamics of interstitial DA and also adaptive or compensatory changes in postsynaptic responsiveness to DA signaling in the course of progressive nigrostriatal degeneration. In particular, we found that failure of DA signaling can occur by different mechanisms at different disease stages. We review these results and discuss their relevance for clinical and translational research, and we draw a number of predictions from our model that might be tested in preclinical experiments. © 2016 The Authors. Movement Disorders published by Wiley Periodicals, Inc. on behalf of International Parkinson and Movement Disorder Society.

The pathological hallmark of Parkinson's disease (PD) is a progressive loss of the mesencephalic dopamine (DA) neurons of the substantia nigra and a resultant depletion of striatal DA fibers.^1^ However, the characteristic motor signs of PD emerge only after substantial nigrostriatal degeneration has occurred, and this in association with changes in a number of components of the extrapyramidal motor system.^2^, ^3^, ^4^ It is therefore presumed that PD manifests only when the extent of striatal dopamine depletion exceeds the tolerance of compensatory or adaptive mechanisms. However, the cause for the ultimate failure of compensation and its relation to a functional threshold for declining DA signaling have not been identified.^4^, ^5^

We have recently developed a mathematical analysis of declining DA signaling and its compensatory adaptations over a broad range of progressive nigrostriatal denervation.^6^ The theoretical analysis was based on a biophysically constrained modeling paradigm^7^, ^8^ where firing patterns of nigrostriatal DA neurons are related to the activation of postsynaptic D1 and D2/3 receptors by explicitly calculating the spatiotemporal dynamics of DA concentrations in a small striatal volume. The model is derived from physiological principles, including vesicular DA release, extracellular diffusion, reuptake, and modulation of neuronal activity by presynaptic and somatodendritic D2‐like autoreceptors. The main insight derived from our previous modeling studies is that synchronized phasic activity of DA neurons provides fast and efficient information transfer to postsynaptic D1‐like and D2‐like receptors. Results from the model have proven consistent with measurements of extracellular DA levels in rodents,^9^, ^10^, ^11^ activation of postsynaptic DA receptors,^12^, ^13^ and behavior.^14^, ^15^, ^16^ As such, the model offers a platform to test the consistency of our knowledge of DA anatomy and normal physiology and how these are affected by drugs or disease.

We therefore applied the model to investigate the DA signal in denervated striatum, asking the following 2 questions: How does denervation influence DA levels? How does this affect signal transduction to postsynaptic targets?

We found that denervation affects extracellular DA levels by 2 mechanisms. At disease stages where remaining innervation is spatially coherent, there is no reduction of average extracellular DA, but a profound reduction of the amplitude of phasic DA signaling. Later, when spatial coherence is lost, there will also be a reduction of extracellular DA. Given this biphasic pattern of changes in the DA signature, we investigated how the activation of postsynaptic neurons might be affected given a certain ability to adapt to these changes in the DA signal. In particular, we asked if there are aspects that evade compensation and could lead to the development of PD. We identified 3 principal mechanisms in which denervation deteriorates the DA signal. Two of these occur in the early and intermediate stages, when denervation solely manifests as reduced phasic signaling. The first effect that occurs is a distortion of DA signaling. Here the translation of dopamine D2/3 receptors regulated signals is skewed such that the temporal signal becomes dominated by few large events at the expense of lost responsiveness to small signals. With progressive denervation, the declining phasic DA signal will eventually become indistinguishable from random fluctuations in tonic DA levels. If postsynaptic DA receptors increase their sensitivity to compensate reduced phasic DA signals, they will eventually also react to random fluctuations in the DA baseline. This second malfunction causes increased disinhibition of D2/3 receptor‐regulated signaling and was relieved in simulations of l‐dopa therapy. Therefore, we propose that this as a candidate mechanism for emerging PD, and we explore it further in the present manuscript. Finally, at advanced stages of denervation, striatal subvolumes completely void of innervation develop, which we identified as the third principal mechanism that distorts DA signaling. Inside fully denervated subvolumes, all DA signaling is lost, and resident neurons of the indirect pathway loose tonic D2 receptor inhibition, as described in classical models of PD. Our model thus provides a new explanation for the onset and early stages of PD but aligns with the classical picture at late stages.

We next discuss our analysis and predictions of the model in greater detail.

## Denervation Increases Heterogeneity of Remaining DA Innervation

**Figure 1:**
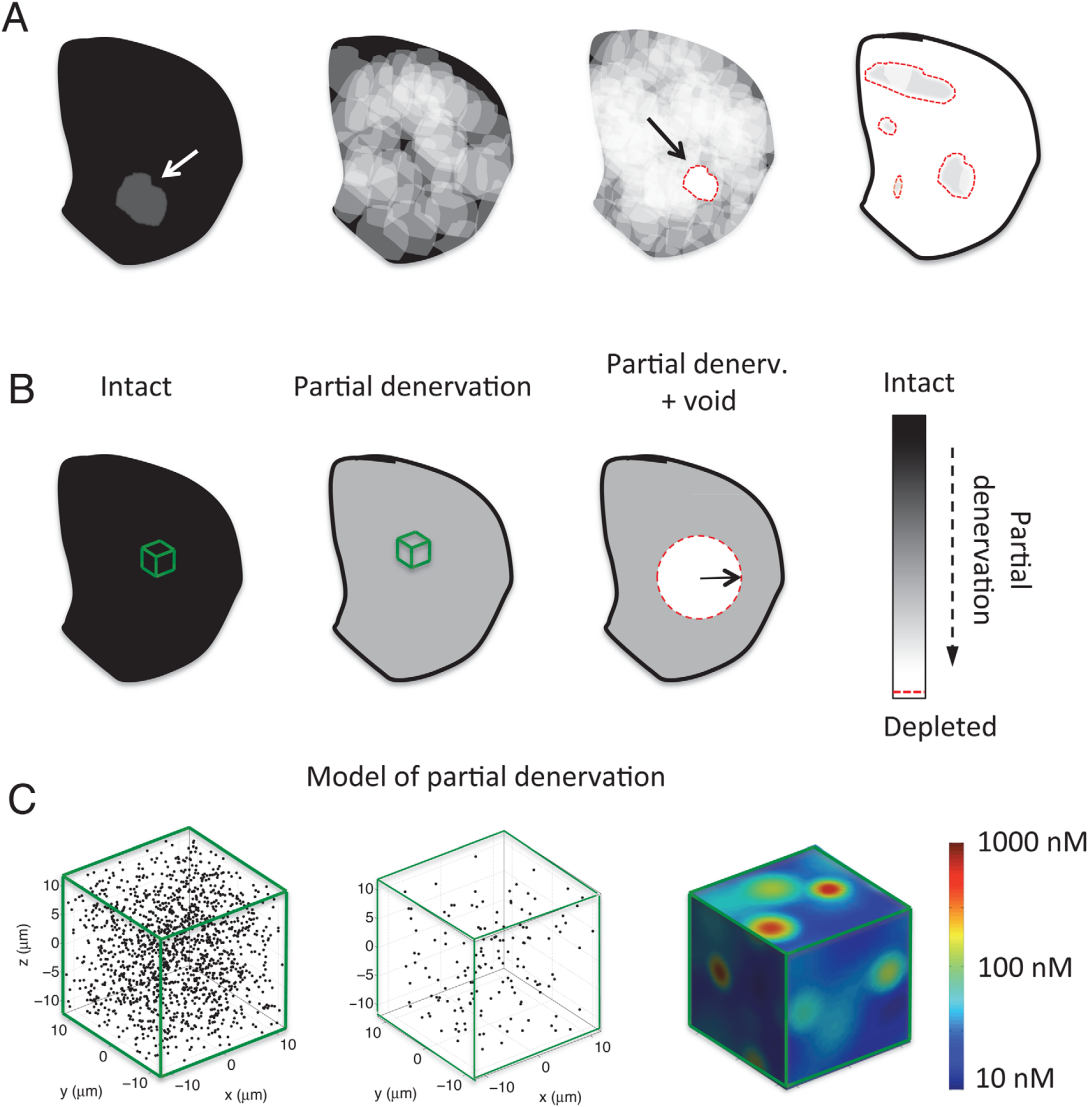
Progressive DA denervation of idealized rodent striatal hemisphere and mathematical idealizations. Black indicates intact innervation, white shows fully depleted areas, and gray scale indicates partial denervation. Red dashed lines indicate boundary of fully denervated regions. **A**: An analysis of progressive innervation (proceeding from left to right) loss of a single DA axon (indicated by arrow), multiple axons, the first appearance of void, and finally a denervation condition with predominant voids. **B**: The mathematical idealizations used in Ref. 6. **C**: The microscale model used for describing partial denervation. Here, the black dots indicate individual DA terminals, that is, the sites of neurotransmitter release and uptake with (proceeding from left to right) intact innervation, 90% denervation, along with the extracellular DA levels during cell firing. [Color figure can be viewed in the online issue, which is available at wileyonlinelibrary.com.]

Nigrostriatal DA neurons form wide axonal arborizations. In the rat, a single DA neuron may influence 2.7% of striatal volume, or roughly 100,000 of postsynaptic neurons. The axonal arbors of individual DA neurons overlap greatly, such that 100 to 200 DA neurons may release DA in proximity to a single postsynaptic neuron.^17^ Thus, with progressive attrition of DA neurons, there will be some threshold where the number of neurons projecting to certain areas is reduced, but every striatal neuron remains within axonal arbor of least 1 DA neuron (Fig. 1A, gray areas), a condition we define as the partially denervated regime. With continued disease progression, striatal volumes fully devoid of innervation emerge (Fig. 1A, white). Our model shows that the DA signal in partially denervated areas differs radically from that when voids appear. This issue is critical because describing the 2 phases of denervation mathematically required different modeling approaches (Fig. 1B,C).

Coherence of innervation also has relevance to innervation patterns in diagnosed patients. For example, in a recent postmortem immunohistochemical examination of brains from patients dying with PD, Kordower and colleagues documented the distribution of remaining DA innervation.^18^ Consistent with early neurochemical analysis,^1^ they found homogeneously distributed 50% loss of DA transporter (DAT) immunoreactivity in cases up to 4 years postdiagnosis. In cases of death more than 4 years postdiagnosis, Kordower and colleagues observed a nearly complete loss of DA innervation, in which prima facie and our model predicts abnormally low extracellular DA concentrations. The immunohistochemical findings showed little evidence for the patchy sparing of innervation depicted in Figure 1. We suppose that the presence of isolated voids may be a relatively transient phenomenon or may be obscured by the sprouting of remaining axons. However, voids may be associated with the rodent intrastriatal 6‐hydroxydopamine (6‐OHDA) lesion model of PD.^19^

Molecular imaging in vivo with PET or single‐photon emission computed tomography cannot capture lesion morphology at a scale less than 1 mm.^20^ Because of resolution issues, symptom severity and disease duration are usually correlated with a mean loss of DA innervation on a cm scale.^21^, ^22^ However, symptoms likely emerge from regions with the highest local denervation. In the following analysis, the stated degree of denervation does not indicate the total DA neuron loss but, rather, the degree to which denervation has affected a localized region.

## DA Signaling With Coherent Innervation

**Figure 2:**
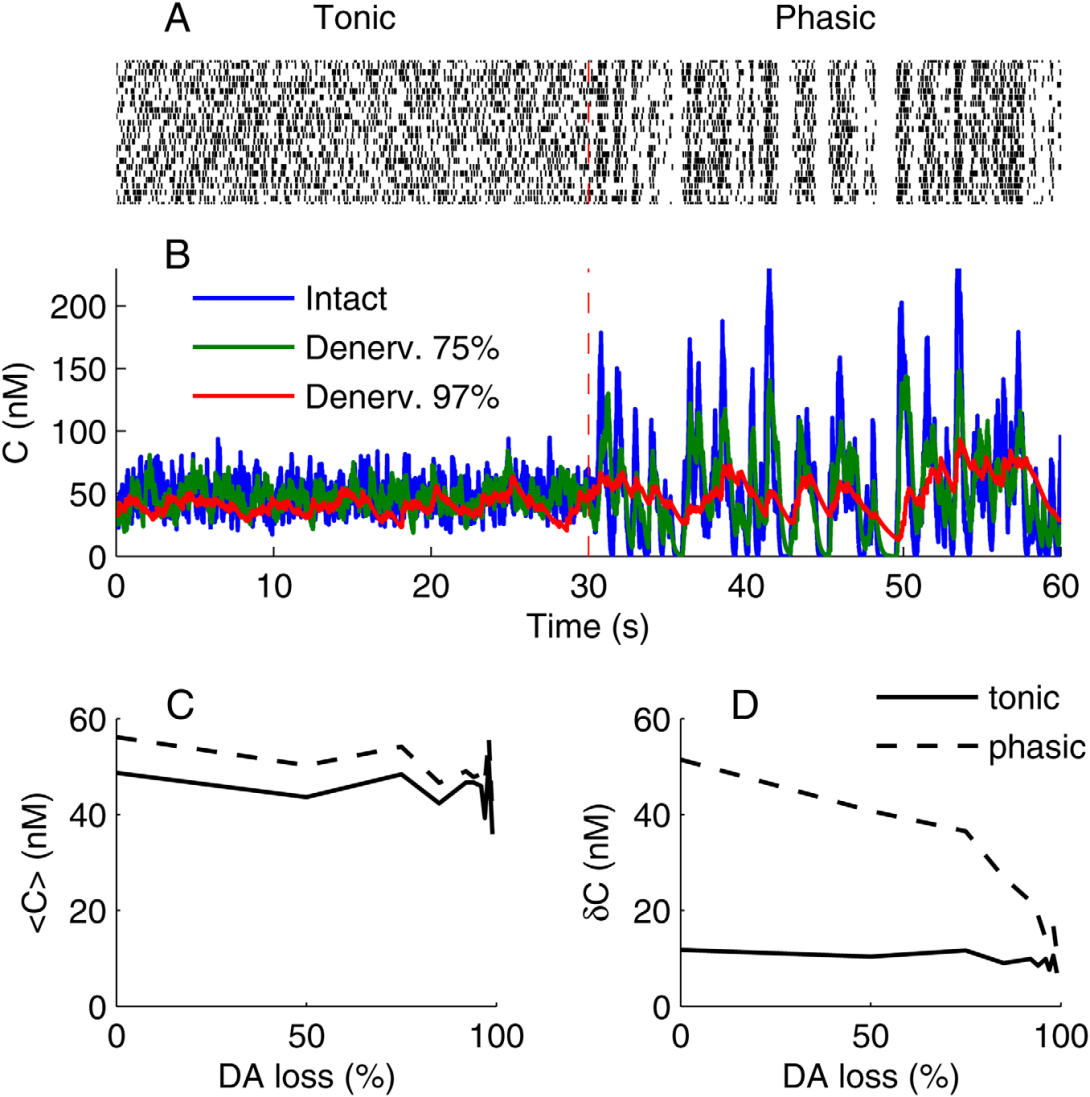
Effects of DA neuron firing pattern on extracellular DA dynamics during denervation. **A**: A raster plot of the cell firing used as input for the model. The vertical dashed line indicates a transition from tonic to phasic firing pattern, in which the mean firing rate (Hz) is unchanged. **B**: The extracellular DA levels with progressive denervation, as indicated by the legend. **C**: The mean extracellular DA concentration with tonic (solid) and phasic cell firing (dashed). **D**: The standard deviation of DA concentration under tonic (solid) and phasic cell firing (dashed). [Color figure can be viewed in the online issue, which is available at wileyonlinelibrary.com.]

Our previous modeling studies have shown that synchronized phasic modulation of the DA firing rate (up or down) provides a strong signal at postsynaptic (D1‐like and D2‐like) receptors. Furthermore, it is known that factors such as external stimuli and genetic or pharmacological manipulations influence the degree of DA neuron burst firing.^23^, ^24^, ^25^ Therefore, we calculated the predicted DA signal using 2 types of spiking patterns: a nonsynchronized firing pattern (*tonic*,^26^ shown in Fig. 2A at *t < * 30 seconds) and a modulated firing pattern with synchronized ∼20 Hz bursts interspersed with random pauses (*phasic*,^27^ shown in Fig. 2A at *t > * 30 seconds). We constrained the mean firing rate to 4 Hz for both firing patterns based on literature findings.

With tonic firing, the mean and standard deviation of the extracellular DA concentration were unaffected by partial denervation (Fig. 2B,C, solid line, tonic; dashed line, phasic). In contrast, the magnitude of phasic DA signaling was strongly attenuated by partial denervation. Not only did DA neuron burst firing lead to less phasic increases in DA levels but also random pauses in DA cell firing lead to attenuated decreases (Fig. 2B, see DA levels for *t* > 30 seconds and dashed line in Fig. 2D). Because the variability in the DA baseline is constant, the ability of postsynaptic neurons to distinguish phasic changes in DA levels from random fluctuations is consequently reduced. At a sufficient state of denervation, the functional phasic DA signal completely disappeared (Fig. 2B, red; Fig. 2D, solid and dashed lines converge).

Observations of reduced phasic DA signals were made by Bergstrom and Garris using fast‐scan cyclic voltammetry in 6‐OHDA lesioned rats^28^, ^29^ and by Howard and colleagues in methamphetamine‐induced denervation.^30^, ^31^ They noted that the steady‐state extracellular DA concentration could remain constant because both release and uptake are reduced to the same extent by denervation, a phenomenon Bergstrom and Garris called “passive stabilization.” Our DA model inherently includes this effect because DA release sites serve as sources and sinks for DA.^8^ Our simulation also included the effects of DA catabolism or nonspecific uptake, but with negligible effect. The continued presence of normal tonic levels of DA is often interpreted as a compensatory effect that precludes declaration of the cardinal motor symptoms of PD.^28^, ^29^, ^32^ However, we find this view oversimplified. To address the functional effects of denervation, one needs also to consider changes in signaling at a systems level, in a model that includes the adaptation of postsynaptic signaling. We argue that homeostatic adaptions in postsynaptic signals can lead to cardinal symptoms of PD despite normal tonic DA levels.

The great majority of neurons resident in striatum are the GABAergic medium spiny striatal projection neurons (MSPNs). This population is of 2 main types: MSPN of the direct pathway express DA D1‐like receptors (D1Rs) and project directly to the internal segment of the globus pallidus (GPi), whereas MSPNs of the indirect pathway express DA D2‐like receptors (D2Rs) and regulate GPi via projections to the external segment and the subthalamic nucleus.^33^ For simplicity, we consider only D1R signal transduction via G_s_, which stimulates downstream production of cyclic adenosine monophosphate (cAMP), and D2R via G_i/o_, which inhibits downstream production of cAMP. The responsiveness of these pathways to DA agonism is highly plastic in response to denervation.^2^, ^3^, ^34^ For the present, we do not attempt to formulate the putative intracellular feedback mechanisms for postsynaptic sensitization. Instead, we take a generalized approach where DA signaling is regulated by 2 parameters: a threshold and a gain. The threshold parameter describes the receptor occupancy that triggers the signaling cascade. We assume G_s_‐coupled signals (D1) to be activated by DA levels higher than the activation threshold, whereas Gi/o‐coupled signals (D2) are activated by DA levels lower than the threshold. The gain parameter describes how much the postsynaptic cascade is activated at a certain deviation relative to the threshold.

**Figure 3:**
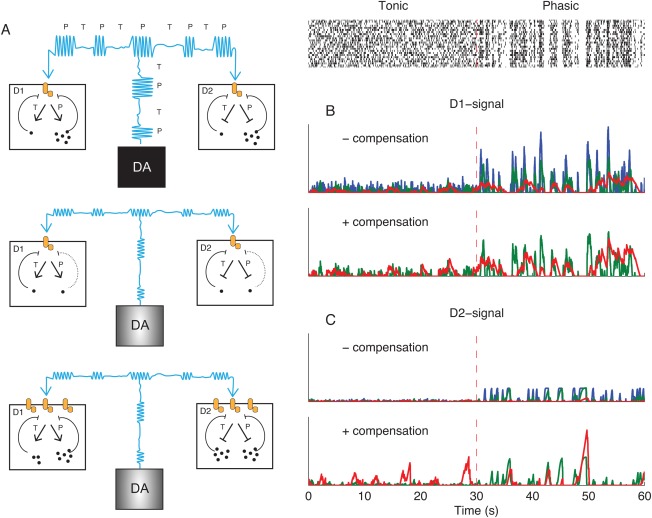
Postsynaptic effects of DA cell firing. **A**: Schematic illustration of hypothetical postsynaptic adaptations to passive stabilization. A, top: DA signals in D1 and D2‐receptor expressing GABAergic medium spiny striatal projection neurons (SPNs) under normal conditions. Cyan indicates the DA level produced by alternation of phasic (P) and tonic (T) DA neuron firing. Dots inside D1‐ and D2‐SPNs represent the products of down‐stream signaling, for example, cAMP levels. These products are mainly produced under phasic DA signals, and we assume that they provide negative feedback on the receptor‐effector unit (orange). A, middle: Partial denervation has reduced the amplitude of phasic DA levels (cyan) and the activation of postsynaptic cascades is reduced. A, bottom: Increasing the gain of postsynaptic receptors phasic signals can compensate reduced phasic signals, but modeling shows increased activation under tonic firing. **B** and **C**: Implementation of adaptive pathways in the computational model. Blue, intact signaling; green, 75% denervated; red, 97% denervated. The raster plot is the same as in Figure 2A. B, upper: D1 receptor mediated signaling without compensation. In the absence of compensation, the postsynaptic activation during phasic firing is decreased by denervation. B, bottom: Denervated D1 signal with phasic amplitude compensation. C: Same as B, but for D2 signaling. [Color figure can be viewed in the online issue, which is available at wileyonlinelibrary.com.]

We set the activation thresholds for D1R and D2R pathways to be equal to the mean binding during tonic firing, and we set the gain to be inversely proportional to the postsynaptic activity evoked by phasic DA cell firing. Biologically, changes in gain could arise from the altered expression of receptors or their coupling to G‐proteins and effectors, whereas changes in activation threshold could arise through the altered stoichiometry of receptors and effectors.^35^ The assumed regulation of gain and threshold may emerge from homeostatic negative feedback (Fig. 3A).^36^

## Compensating the Loss of Phasic Signals Leads to Aberrant Disinhibition of Postsynaptic Pathways

Postsynaptic pathways would normally have low activation under tonic DA neuron firing and high activation under phasic firing (Fig. 3B,C, blue). The compensation for reduced phasic amplitude occurring in the passive stabilized regime is an increased gain of postsynaptic signals. This could restore a functional DA signal so long as denervation was less than 50%. However, with increasing denervation, the D2R‐regulated signal was skewed such that short pauses activated the indirect pathway less, whereas long pauses evoked disproportionally large postsynaptic responses (Fig. 3C, green). When denervation exceeded 70% in our model, the contrast between baseline dopamine and the functional signal was lost. Beyond this point, the gain is so high that the postsynaptic cascade is persistently activated by random fluctuations in the DA baseline (Fig. 3C). Although the model predicts that random activation may occur in D1R‐ and D2R‐regulated signals, we found that D2R‐regulated pathways proved more susceptible than D1R‐regulated signals. One reason for this is that the pauses in firing have a lower signal‐to‐noise ratio than do bursts; the firing rate can only be reduced from the 4 Hz baseline to nil, whereas typical bursts increase the firing rate by 15 Hz.

Our model thus predicts that disinhibition of D2R‐regulated pathways, often considered a cardinal feature of clinical PD, occurs in the presence of passively stabilized DA levels arising from extensive but coherent nigrostriatal denervation. An obvious test of the heuristic value of our model would be to consider if aberrant signals are moderated by l‐dopa therapy, simulated by increasing vesicular release quanta.^37^ However, the increased baseline under l‐dopa will also have consequences for gain and threshold of postsynaptic signals. Therefore, we simulated 2 conditions of postsynaptic adaptation: In simulations of acute l‐dopa treatment, synaptic signals were adapted to the predrug denervated state. In simulations of long‐term l‐dopa treatment, the gain and activation threshold were adjusted to the postdrug DA signal. In both cases, l‐dopa proved to have only a small effect on DA signaling in the intact striatum (which matches the experience that l‐dopa is without great effect in healthy individuals). The aberrant D2R signal was reduced in simulations of acute l‐dopa challenge. However, further simulations also showed that in the long term, this presumably beneficial effect of l‐dopa is lost as a result of postsynaptic homeostasis. Interestingly, we had the opposite results for the D1R‐regulated pathway. Here, aberrant D1R signals occurred with acute l‐dopa treatment but were eventually attenuated by postsynaptic adaptation. The effect of direct DA agonists, such as apomorphine or pramipexole, was not investigated, but we expect that their effect on aberrant D2 signals would be similar to those of l‐dopa. However, unlike l‐dopa, a direct agonist would also affect areas with intact DA innervation.

## Late‐Stage PD

In our model, passive stabilization breaks down when DA innervation loses its spatial coherence. Inside striatal voids there can be neither DA release nor DAT‐mediated reuptake. Consequently, the extracellular DA level within voids depends on the balance between diffusional influx from distal sources and enzymatic degradation or DA reuptake in non‐DA elements such as glia. Because of diffusion limits, the DA concentration declines with increasing size of the voids; we calculate that within voids greater than 500 µm in diameter, DA levels will be tonically 20% of normal. The time course of the effects of late‐stage denervation on postsynaptic signaling cascades were deemed too complex to be emulated or quantified in our model analysis. However, we expect this regime to be in agreement with classical models of PD.^38^

All neurobiological models necessarily entail some simplification. We simulated the effects of DA neuron attrition but assumed that the remaining DA neurons were themselves unaltered (except for autoreceptor‐mediated factors). However, high levels of intracellular α‐synuclein may inherently reduce the firing rate and hamper DA release in surviving neurons.^39^ Such effects would contribute to reduced extracellular DA levels and complicate the required postsynaptic adaptations. Other factors such as axonal sprouting, regulation of DA synthesis, and DA reuptake are also absent in the present model. However, presynaptic adaptation, if mediated by presynaptic or somatodendritic autoreceptors, would be expected to occur late and to be mostly pronounced at the boundary between partially denervated and fully depleted areas, that is, where there is a gradient of tonic DA concentration.

## Predictions for Translational and Clinical Research

In the following sections, we present predictions arising from the theoretical analysis. First, we discuss the predicted relationship between lesion and turning behavior in rodents. Second, we predict how certain observations in rodents may bear relevance for detecting subclinical PD in humans.

### Relationship Between Lesion and Turning Behavior in Rodents

The rotation model is a classical rodent model of PD,^40^ often used to test potential therapies. Here, infusion of the toxin 6‐OHDA in one medial forebrain bundle creates a unilateral lesion the nigrostriatal pathway. After a period of adaptation, challenge with amphetamine, cocaine, or direct DA agonists provoke turning behavior either toward the side of the lesion (ipsiversion) or away from the side of the lesion (contraversion). Our mathematical model can be used to make qualitative predictions of the magnitude of D1R‐ and D2R‐regulated signaling in each hemisphere of hemiparkinsonian rats. This can be used to make direct predictions about the direction of the rotation relative to lesion. We assume that activation of D1R stimulates the direct pathway, that activation of D2R inhibits the indirect pathway, and that strong activation of the direct pathway and inhibition of the indirect pathway promotes locomotion on the contralateral body side.^41^

**Figure 4:**
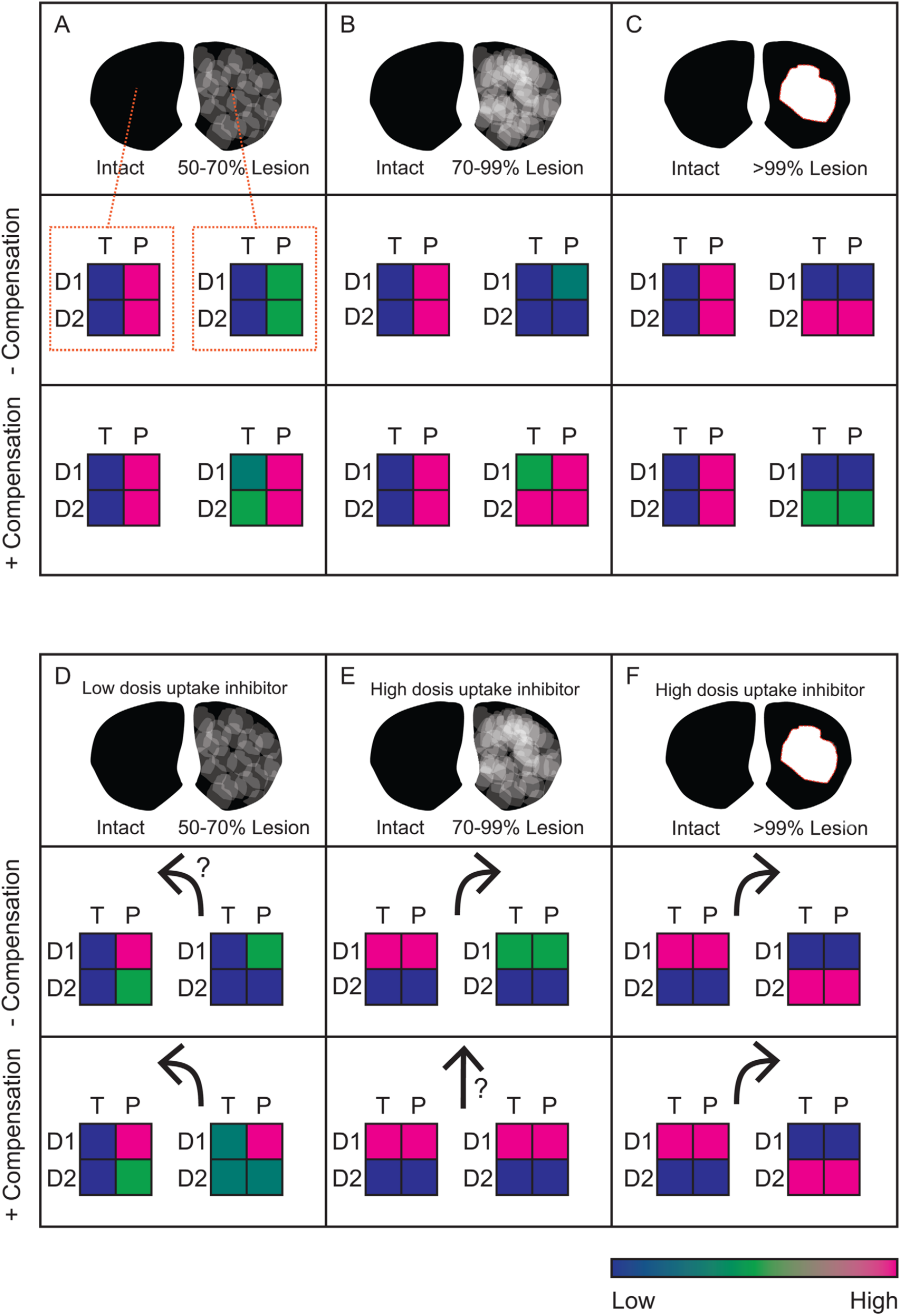
Overview of predictions for unilateral lesions from the theoretical analysis in Dreyer 6. Striatal sections with unilateral lesions are divided into 3 cases: low coherent (**A, D**), high coherent (**B, E**), and void (**C, F**). Activation level of D1‐ or D2‐regulated signals (D1 and D2, respectively) under tonic (T) and phasic (P) signaling are indicated by the color scale. A, B, and C are under normal conditions, whereas D is under low dopamine uptake inhibition. E and F are under high‐dose psychostimulant challenge. Arrows indicate predicted animal rotation direction (relative to the lesion). [Color figure can be viewed in the online issue, which is available at wileyonlinelibrary.com.]

We restrict our analysis to the 3 major conditions identified in an earlier work.^6^ Low denervation, which skews D2R signaling but without random aberrant signaling (<70% denervation, Fig. 4A), excess coherent denervation, where random postsynaptic activation may lead to the first motor manifestations of PD (Fig. 4B), and the presence of voids with complete denervation (Fig. 4C). For each degree of denervation, we estimated the average activation of D1R‐ or D2R‐regulated signals under tonic (T) and phasic (P) signaling separately for the intact and lesioned hemispheres and for the cases of acute and compensated hemilesions (−compensation and +compensation, respectively). The color code in Figure 4A‐C schematically recapitulates our discussion above: In the intact hemisphere, D1R‐ and D2R‐regulated signaling is low under tonic cell firing, but high under phasic firing. Depending on the degree of denervation, the phasic signals are reduced without compensation or increased with compensation. Activity under tonic firing occurs in both pathways when a lack of phasic signaling is adequately compensated.

In an earlier study,^6^ we first modeled how expected signals in the condition of low denervation (50% to 70%) would be altered by DA uptake inhibition. Our model predicts that D2R receptors saturate by low uptake inhibition (equivalent to 5–10 mg/kg i.p. cocaine^8^), effectively quenching phasic signaling. The resulting inhibition of the indirect pathway in the lesioned hemisphere may manifest in a contraversive motor response (Fig. 4D). Examples of such contraversive turning under mild unilateral lesions are well documented in rats.^19^, ^42^, ^43^

Rotations in rodents with partial lesions are also studied with higher doses of amphetamines.^44^, ^45^, ^46^ However, the D2R differences discussed previously are less important. With a strong amphetamine challenge, reuptake is reduced and counter transport by facilitated exchange‐diffusion so pronounced that phasic D2R signaling is quenched in both hemispheres. Therefore, we compare the activation of D1R pathways in each hemisphere in relation to the extent of rotation (Fig. 4E,F). Knowing that a high dose of amphetamine results in a reverse flux of DA from terminals,^47^ we expect that extracellular DA should then increase at a rate roughly proportional to the remaining innervation in each hemisphere. With an acute lesion, prior to postsynaptic adaptations, we can expect a stronger activation of D1Rs in the intact hemisphere, leading to ipsiversion. However, increasing postsynaptic gain in the lesioned hemisphere will tend to reduce rotation. These opposing effects would possibly cancel out when adaptation is fully developed (Fig. 4E). As such, turning behavior under these conditions may not reflect the degree by which the animal is affected by the lesion in drug‐free conditions.

In the case of an extensive unilateral lesion, the response to amphetamines may be understood more intuitively; here DA release occurs only in the intact hemisphere where full and concerted activation of D1Rs and D2Rs will both promote strong ipsiversive turning (Fig. 4F).^40^

### Enhanced Sensitivity for Methylphenidate for Patients With Subclinical PD?

The prediction of contraversive rotations in rodents under a low dose of uptake inhibition (Fig. 4D) may have relevance for the clinical detection of presymptomatic PD, often presenting with a vague complaint of fatigue. Our model predicts that D2R‐regulated behavior will be more susceptible to DA uptake inhibition at low denervation. In particular, our model predicts that patients with subclinical loss of DA neurons will exhibit increased sensitivity to the locomotor stimulant effects of DA uptake inhibitors such as methylphenidate. Therefore, we expect that methylphenidate will provoke locomotor effects at lower doses in patients experiencing a subclinical loss of DA neurons than in healthy individuals. Demonstrating this phenomenon could potentially provide a test for subclinical DA loss.

We have attempted to reformulate the textbook box‐and‐arrow diagram of basal ganglia circuitry in PD and draw from our computational model more subtle predictions about the pre‐ and postsynaptic adaptations that are expected to occur, and at which disease stage compensation may break down. Our idealized mathematical approach provides a classification of DA signals based on anatomical coherence and accommodates known features of DA physiology, such as tonic and phasic DA cell firing, large‐scale morphology of denervation patterns, and postsynaptic adaptations to changes in both tonic and phasic DA signals. In particular, we find somewhat counterintuitive results for early stages of denervation, which may reflect the real complexity of adaptive changes in the face of progressive loss of the striatal DA signal. We anticipate that more refined models should yield additional insights, such as prediction of motor side effects of medication.

## Author Roles

(1) Research Project: A. Conception, B. Organization, C. Execution; (2) Statistical Analysis: A. Design, B. Execution, C. Review and Critique; (3) Manuscript: A. Writing of the First Draft, B. Review and Critique.

J.K.D.: 1A, 1B, 1C, 3A, 3B

C.A.N.: 1C, 3A, 3B

**Full financial disclosures of all authors for the past year**: J.K.D. receives financial support from the Lundbeck Foundation, University of Copenhagen 2016 Excellence Programme, for Interdisciplinary Research, and the NIH Brian Initiative Award R24 MH106083.

C.A.N receives financial support from the Siemens Foundation Denmark, the Lundbeck Foundation, and the Augustinus Foundation.

## References

[1] Kish SJ , Shannak K , Hornykiewicz O . Uneven pattern of dopamine loss in the striatum of patients with idiopathic Parkinsons‐disease—pathophysiologic and clinical implications. New Engl J Med 1988;318(14):876–880. 3352672 10.1056/NEJM198804073181402

[2] Gerfen CR . D1 dopamine receptor supersensitivity in the dopamine‐depleted striatum animal model of Parkinson's disease. Neuroscientist 2003;9(6):455–462. 14678578 10.1177/1073858403255839

[3] Bezard E , Gross CE , Brotchie JM . Presymptomatic compensation in Parkinson's disease is not dopamine‐mediated. Trends Neurosci 2003;26(4):215–221. 12689773 10.1016/S0166-2236(03)00038-9

[4] Arkadir D , Bergman H , Fahn S . Redundant dopaminergic activity may enable compensatory axonal sprouting in Parkinson disease. Neurology 2014;82(12):1093–1098. 24663231 10.1212/WNL.0000000000000243

[5] Obeso JA , Rodriguez‐Oroz MC , Lanciego JL , Rodriguez Diaz M . How does Parkinson's disease begin? The role of compensatory mechanisms. Trends Neurosci 2004;27(3):125–127; author reply 127‐128. 15036875 10.1016/j.tins.2003.12.006

[6] Dreyer JK . Three mechanisms by which striatal denervation causes breakdown of dopamine signaling. J Neurosci 2014;34(37):12444–12456. 25209283 10.1523/JNEUROSCI.1458-14.2014 PMC6615501

[7] Dreyer JK , Herrik KF , Berg RW , Hounsgaard JD . Influence of phasic and tonic dopamine release on receptor activation. J Neurosci 2010;30(42):14273–14283. 20962248 10.1523/JNEUROSCI.1894-10.2010 PMC6634758

[8] Dreyer JK , Hounsgaard J . Mathematical model of dopamine autoreceptors and uptake inhibitors and their influence on tonic and phasic dopamine signaling. J Neurophysiol 2013;109(1):171–182. 23054599 10.1152/jn.00502.2012

[9] Floresco SB , West AR , Ash B , Moore H , Grace AA . Afferent modulation of dopamine neuron firing differentially regulates tonic and phasic dopamine transmission. Nat Neurosci 2003;6(9):968–973. 12897785 10.1038/nn1103

[10] Venton BJ , Zhang H , Garris PA , Phillips PE , Sulzer D , Wightman RM . Real‐time decoding of dopamine concentration changes in the caudate‐putamen during tonic and phasic firing. J Neurochem 2003;87(5):1284–1295. 14622108 10.1046/j.1471-4159.2003.02109.x

[11] Dreyer JK , Vander Weele CM , Lovic V , Aragona BJ . Functionally distinct dopamine signals in nucleus accumbens core and shell in the freely moving rat. J Neurosci 2016;36(1):98–112. 26740653 10.1523/JNEUROSCI.2326-15.2016 PMC6601791

[12] Bertran‐Gonzalez J , Bosch C , Maroteaux M , et al. Opposing patterns of signaling activation in dopamine D1 and D2 receptor‐expressing striatal neurons in response to cocaine and haloperidol. J Neurosci 2008;28(22):5671–5685. 18509028 10.1523/JNEUROSCI.1039-08.2008 PMC6670792

[13] Svenningsson P , Lindskog M , Ledent C , et al. Regulation of the phosphorylation of the dopamine‐ and cAMP‐regulated phosphoprotein of 32 kDa in vivo by dopamine D1, dopamine D2, and adenosine A2A receptors. Proc Natl Acad Sci U S A 2000;97(4):1856–1860. 10677546 10.1073/pnas.97.4.1856 PMC26526

[14] Porter‐Stransky KA , Seiler JL , Day JJ , Aragona BJ . Development of behavioral preferences for the optimal choice following unexpected reward omission is mediated by a reduction of D2‐like receptor tone in the nucleus accumbens. Eur J Neurosci 2013;38(4):2572–2588. 23692625 10.1111/ejn.12253

[15] Cox SM , Frank MJ , Larcher K , et al. Striatal D1 and D2 signaling differentially predict learning from positive and negative outcomes. Neuroimage 2015;109:95–101. 25562824 10.1016/j.neuroimage.2014.12.070

[16] Hikosaka O . Basal ganglia mechanisms of reward‐oriented eye movement. Ann N Y Acad Sci 2007;1104:229–249. 17360800 10.1196/annals.1390.012

[17] Matsuda W , Furuta T , Nakamura KC , et al. Single nigrostriatal dopaminergic neurons form widely spread and highly dense axonal arborizations in the neostriatum. J Neurosci 2009;29(2):444–453. 19144844 10.1523/JNEUROSCI.4029-08.2009 PMC6664950

[18] Kordower JH , Olanow CW , Dodiya HB , et al. Disease duration and the integrity of the nigrostriatal system in Parkinson's disease. Brain 2013;136(pt 8):2419–2431. 23884810 10.1093/brain/awt192 PMC3722357

[19] Labandeira‐Garcia JL , Rozas G , Lopezmartin E , Liste I , Guerra MJ . Time course of striatal changes induced by 6‐hydroxydopamine lesion of the nigrostriatal pathway, as studied by combined evaluation of rotational behaviour and striatal Fos expression. Exp Brain Res 1996;108(1):69–84. 8721156 10.1007/BF00242905

[20] Rahmim A , Zaidi H . PET versus SPECT: strengths, limitations and challenges. Nucl Med Commun 2008;29(3):193–207. 18349789 10.1097/MNM.0b013e3282f3a515

[21] Zhao YJ , Wee HL , Chan YH , et al. Progression of Parkinson's disease as evaluated by Hoehn and Yahr stage transition times. Mov Disord 2010;25(6):710–716. 20213822 10.1002/mds.22875

[22] Knudsen GM , Karlsborg M , Thomsen G , et al. Imaging of dopamine transporters and D2 receptors in patients with Parkinson's disease and multiple system atrophy. Eur J Nucl Med Mol Imaging 2004;31(12):1631–1638. 15583914 10.1007/s00259-004-1578-x

[23] Valenti O , Lodge DJ , Grace AA . Aversive stimuli alter ventral tegmental area dopamine neuron activity via a common action in the ventral hippocampus. J Neurosci 2011;31(11):4280–4289. 21411669 10.1523/JNEUROSCI.5310-10.2011 PMC3066094

[24] Herrik KF , Christophersen P , Shepard PD . Pharmacological modulation of the gating properties of small conductance Ca2+‐activated K + channels alters the firing pattern of dopamine neurons in vivo. J Neurophysiol 2010;104(3):1726–1735. 20660424 10.1152/jn.01126.2009

[25] Soden ME , Jones GL , Sanford CA , et al. Disruption of dopamine neuron activity pattern regulation through selective expression of a human KCNN3 mutation. Neuron 2013;80(4):997–1009. 24206670 10.1016/j.neuron.2013.07.044 PMC3840077

[26] Grace AA , Bunney BS . The control of firing pattern in nigral dopamine neurons: single spike firing. J Neurosci 1984;4(11):2866–2876. 6150070 10.1523/JNEUROSCI.04-11-02866.1984 PMC6564731

[27] Grace AA , Bunney BS . The control of firing pattern in nigral dopamine neurons: burst firing. J Neurosci 1984;4(11):2877–2890. 6150071 10.1523/JNEUROSCI.04-11-02877.1984 PMC6564720

[28] Bergstrom BP , Garris PA . “Passive stabilization” of striatal extracellular dopamine across the lesion spectrum encompassing the presymptomatic phase of Parkinson's disease: a voltammetric study in the 6‐OHDA‐lesioned rat. J Neurochem 2003;87(5):1224–1236. 14622102 10.1046/j.1471-4159.2003.02104.x

[29] Bergstrom BP , Sanberg SG , Andersson M , Mithyantha J , Carroll FI , Garris PA . Functional reorganization of the presynaptic dopaminergic terminal in parkinsonism. Neuroscience 2011;193:310–322. 21787843 10.1016/j.neuroscience.2011.07.029 PMC3171576

[30] Howard CD , Keefe KA , Garris PA , Daberkow DP . Methamphetamine neurotoxicity decreases phasic, but not tonic, dopaminergic signaling in the rat striatum. J Neurochem 2011;118(4):668–676. 21668447 10.1111/j.1471-4159.2011.07342.x PMC3149871

[31] Howard CD , Pastuzyn ED , Barker‐Haliski ML , Garris PA , Keefe KA . Phasic‐like stimulation of the medial forebrain bundle augments striatal gene expression despite methamphetamine‐induced partial dopamine denervation. J Neurochem 2013;125(4):555–565. 23480199 10.1111/jnc.12234 PMC3640634

[32] Reed MC , Best JA , Nijhout HF . Passive and active stabilization of dopamine in the striatum. Biosci Hypotheses 2009;2:240–244.

[33] Surmeier DJ , Ding J , Day M , Wang ZF , Shen WX . D1 and D2 dopamine‐receptor modulation of striatal glutamatergic signaling in striatal medium spiny neurons. Trends Neurosci 2007;30(5):228–235. 17408758 10.1016/j.tins.2007.03.008

[34] Gerfen CR . Molecular effects of dopamine on striatal‐projection pathways. Trends Neurosci 2000;23(10 suppl):S64–70. 11052222 10.1016/s1471-1931(00)00019-7

[35] Kenakin T . New concepts in pharmacological efficacy at 7TM receptors: IUPHAR review 2. Br J Pharmacol 2013;168(3):554–575. 22994528 10.1111/j.1476-5381.2012.02223.x PMC3579279

[36] Turrigiano G . Homeostatic signaling: the positive side of negative feedback. Curr Opin Neurobiol 2007;17(3):318–324. 17451937 10.1016/j.conb.2007.04.004

[37] Pothos EN , Davila V , Sulzer D . Presynaptic recording of quanta from midbrain dopamine neurons and modulation of the quantal size. J Neurosci 1998;18(11):4106–4118. 9592091 10.1523/JNEUROSCI.18-11-04106.1998 PMC6792796

[38] Lewis SJ , Caldwell MA , Barker RA . Modern therapeutic approaches in Parkinson's disease. Expert Rev Mol Med 2003;5(10):1–20. 14987395 10.1017/S1462399403006008

[39] Janezic S , Threlfell S , Dodson PD , et al. Deficits in dopaminergic transmission precede neuron loss and dysfunction in a new Parkinson model. Proc Natl Acad Sci U S A 2013;110(42):E4016–E4025. 24082145 10.1073/pnas.1309143110 PMC3801069

[40] Ungerstedt U , Arbuthnott GW . Quantitative recording of rotational behavior in rats after 6‐hydroxy‐dopamine lesions of the nigrostriatal dopamine system. Brain Res 1970;24(3):485–493. 5494536 10.1016/0006-8993(70)90187-3

[41] Kravitz AV , Freeze BS , Parker PR , et al. Regulation of parkinsonian motor behaviours by optogenetic control of basal ganglia circuitry. Nature 2010;466(7306):622–626. 20613723 10.1038/nature09159 PMC3552484

[42] Paquette MA , Marsh ST , Hutchings JE , Castaneda E . Amphetamine‐evoked rotation requires newly synthesized dopamine at 14 days but not 1 day after intranigral 6‐OHDA and is consistently dissociated from sensorimotor behavior. Behav Brain Res 2009;200(1):197–207. 19378464 10.1016/j.bbr.2009.01.017

[43] Robinson TE , Noordhoorn M , Chan EM , Mocsary Z , Camp DM , Whishaw IQ . Relationship between asymmetries in striatal dopamine release and the direction of amphetamine‐induced rotation during the first week following a unilateral 6‐OHDA lesion of the substantia nigra. Synapse 1994;17(1):16–25. 8042143 10.1002/syn.890170103

[44] Truong L , Allbutt H , Kassiou M , Henderson JM . Developing a preclinical model of Parkinson's disease: a study of behaviour in rats with graded 6‐OHDA lesions. Behav Brain Res 2006;169(1):1–9. 16413939 10.1016/j.bbr.2005.11.026

[45] Boix J , Padel T , Paul G . A partial lesion model of Parkinson's disease in mice—characterization of a 6‐OHDA‐induced medial forebrain bundle lesion. Behav Brain Res 2015;284:196–206. 25698603 10.1016/j.bbr.2015.01.053

[46] Heuer A , Smith GA , Lelos MJ , Lane EL , Dunnett SB . Unilateral nigrostriatal 6‐hydroxydopamine lesions in mice I: motor impairments identify extent of dopamine depletion at three different lesion sites. Behav Brain Res 2012;228(1):30–43. 22146593 10.1016/j.bbr.2011.11.027

[47] Sulzer D . How addictive drugs disrupt presynaptic dopamine neurotransmission. Neuron 2011;69(4):628–649. 21338876 10.1016/j.neuron.2011.02.010 PMC3065181

